# Glyceroglycolipids in marine algae: A review of their pharmacological activity

**DOI:** 10.3389/fphar.2022.1008797

**Published:** 2022-10-21

**Authors:** Sha-sha Guo, Zhen-guo Wang

**Affiliations:** ^1^ Key Laboratory of Theory of TCM, Ministry of Education of China, Shandong University of Traditional Chinese Medicine, Jinan, China; ^2^ Institute of Traditional Chinese Medicine Literature and Culture, Shandong University of Traditional Chinese Medicine, Jinan, China

**Keywords:** glyceroglycolipids, source, structure, pharmacological, marine algae

## Abstract

Glyceroglycolipids are major metabolites of marine algae and have a wide range of applications in medicine, cosmetics, and chemistry research fields. They are located on the cell surface membranes. Together with glycoproteins and glycosaminoglycans, known as the glycocalyx, they play critical roles in multiple cellular functions and signal transduction and have several biological properties such as anti-oxidant and anti-inflammatory properties, anti-viral activity, and anti-tumor immunity. This article focused on the sources and pharmacological effects of glyceroglycolipids, which are naturally present in various marine algae, including planktonic algae and benthic algae, with the aim to highlight the promising potential of glyceroglycolipids in clinical treatment.

## 1 Introduction

Marine algae are photosynthetic plants with energy and nutritional value in the marine ecosystem. Although they are at the bottom of the food chain, they account for more than half of the biodiversity of the aquatic environment. In addition, marine algae represent a rich source of bioactive compounds and secondary metabolites (Saadaoui et al., 2020; Cepas et al., 2021). Based on differences in pigmentation, cell structure, reproductive method, and reproductive organ structure, there are about 27,000 species of marine algae and can be divided into planktonic algae, including Cyanobacteria, Chlamydomonas, Dinophyta, prochlorophytes, Euglena, xanthophyll, Cryptophyta, and diatoms, and benthic algae, including Chlorophyta, pheophytin, and Rhodophyta (Lordan et al., 2011; Guiry et al., 2021; Mandalka et al., 2022). Over the past decades, marine algae have been considered a major constituent for drug discovery. The massive diversification of marine algae in the marine ecosystem serves as a reservoir for a wide range of natural compounds (Agatonovic-Kustrin et al., 2018; Bharadwaj et al., 2022). Presently, marine algae are being researched as sources of food, medicine, cosmetics, fertilizer, fodder, and bioenergy (Zhang et al., 2022). However, the development and utilization of glyceroglycolipids in marine algae have been limited due to their low natural abundance.

**FIGURE 1:**
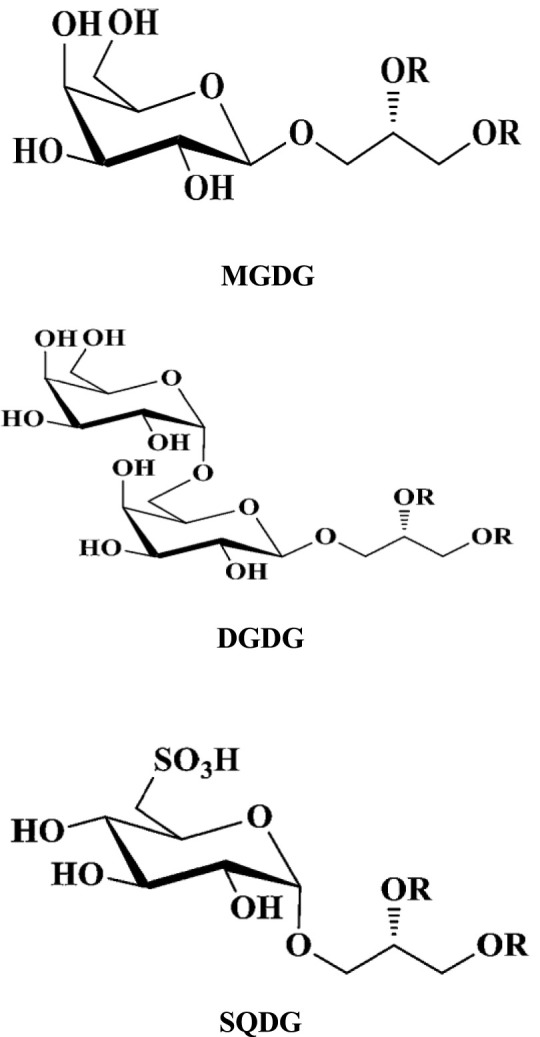
Basic structures of glyceroglycolipids.

Glyceroglycolipids have recently received increasing attention due to their anti-bacterial, anti-viral, anti-tumor, and anti-inflammatory activities (Hölzl and Dörmann, 2007; Michaud et al., 2017; Cepas et al., 2021). They are especially abundant in marine algae (Domingues and Calado, 2022) and can be divided into the following three main categories according to their chemical structures and types of glycosyl and acyl structures: monogalactosyl-diacylglycerols (MGDG), digalactosyl-diacylglycerol (DGDG), and sulfoquinovosyl-diacylglycerol (SQDG) (Marcolongo et al., 2006; Zhang et al., 2014) (Figure 1). Glyceroglycolipids constitute more than half of the total lipids (including fatty acids, glycerolipids, glycerophospholipids, sphingolipids, sterol lipids, saccharolipids, and polyketides). Glyceroglycolipids have several beneficial health effects, including their anti-tumor immunity, anti-oxidant and anti-inflammatory properties, and anti-viral activity (Morimoto et al., 1995; Loya et al., 1998). However, due to the low natural abundance of glyceroglycolipids in marine algae and the difficulty of separation, their development and utilization are subjected to certain restrictions.

In the past few years, there has been increasing interest in researching the benefits of natural pharmacological products in treating various diseases. This review provides a comprehensive overview of glyceroglycolipid libraries obtained from Web of Science, SciFinder, Medline, and other databases. To have an in-depth understanding of the glyceroglycolipids derived from marine algae and highlight their potential and prospects in biology, we summarized the literature on the source, structure, and pharmacology of glyceroglycolipids in marine organisms.

## 2 Methods and summary

### 2.1 Quality appraisal

The quality appraisal of articles was performed in phase three of the PRISMA flowchart (eligibility check) to ensure the quality of the included articles. Relevant information on the marine algae was obtained from scientific online databases such as Google Scholar, PubMed, and Web of Science. Additional information was derived from other literature sources (e.g., Chinese Pharmacopoeia 2020 edition, Chinese herbal classic books, PhD and MSc thesis, etc.). The search process was mainly based on keyword search, with the main keywords being marine algae, glyceroglycolipids, antitumor, antibacterial, and antiviral, etc., for which they were searched individually or in combination. The scope of the literature search was focused on literature reports between 2012 and 2022. Some of these studies were not reported in the last decade, and we extended the search to earlier literature.

**FIGURE 2:**
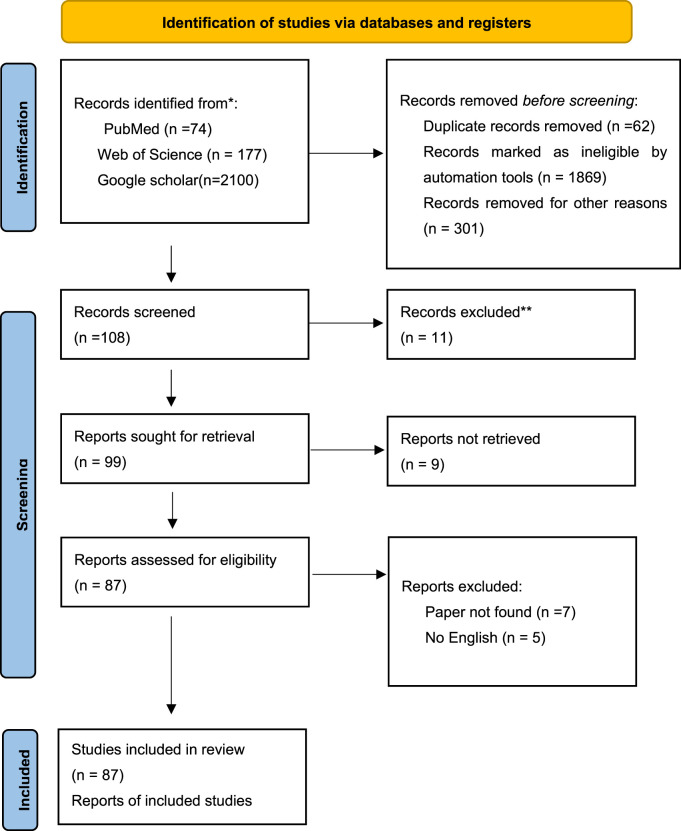
Flow diagram for synergy model review.

The initial search resulted in 2,351 articles. Then, those identified as duplicate articles, editorials, letters, or basic science studies were excluded, and 99 full-text articles were identified. With detailed analysis, we excluded 12 full-text articles (5 non-English; 7 could not be found). A total of 87 full-text articles were found to be relevant for the systematic review (Figure 2).

### 2.2 Source and structure of glyceroglycolipids in marine algae

Glyceroglycolipids are especially abundant in marine algae and exhibit a glycerol backbone that anchors one or two acyl chains esterified at the sn-1 and sn-2 positions and a sugar group attached at the sn-3 position in a β-anomeric linkage (Demé et al., 2014; Hoyo et al., 2016). MGDG, DGDG, and SQDG are the most common glyceroglycolipids, which differ mainly in their fatty acid chain. The saturation and chain length of the fatty acid chains influence the biological activity of glyceroglycolipids. The former two glyceroglycolipids are neutral glycolipids, and the latter one is anionic sulfolipids (Alves et al., 2020; Chen and Wang, 2021). For the broad application of marine algae lipids, it is crucial to identify marine algae materials rich in glyceroglycolipids. Table 1 summarizes the form of fatty acids derived from glycerol in diverse marine algae sources, from which C16 and C18 can be seen as the main acyl fatty acid chains of glycerol glycolipids. According to some research, glycoglycerolipid biosynthesis mainly occurs via two routes: the prokaryotic pathway, which generates glyceroglycolipids with an sn-2 C16 acyl group, and the eukaryotic pathway, which generates glyceroglycolipids with an sn-2 C18 acyl group (Heinz and Roughan, 1983). Several studies have shown that glyceroglycolipids from marine algae have significant roles in connecting structural characteristics and pharmacological activities.

**TABLE 1:** Fatty acids of glyceroglycolipids in several marine algae species.

Species name	FA of the acyl part of glyceroglycolipids (sn-1/sn-2) or (total FA)	Ref
**Chlorophyta**		
*Caulerpa taxifolia*	**MGDG**: (C16:3/C18:3, C18:3/C16:3)	Mancini et al. (1998)
*Chlorella sorokiniana*	**MGDG, DGDG, SQDG:** (C16:0, C16:1n-7, C16:3n-3, C18:0, C18:1n-9, C18:2n-6, C18:3n-3)	Guschina et al. (2020)
*Ulva armoricana*	**MGDG:** C14:0/C16:1n-5; **DGDG:** C14:0/C18:3n-3	Kendel et al. (2015)
*Klebsormidium flaccidum var. zivo*	**MGDG: (C16:4/C18:3, C16:3/C18:3)**	Qiu et al. (2020)
	**DGDG: (C16:4/C18:3, C16:3/C18:3)**	
*Ulvella lens*	**MGDG** (16:0/16:1n-9/16:1n-7/16:2n-6/16:4n-3/18:2n-6/18:3n-3)	Takahashi et al. (2002)
	**DGDG** (14:0/16:0/16:2n-6/16:4n-3/18:2n-6/18:3n-3)	
	**SQDG** (14:0/16:0/18:1n-7/18:2n-6/18:3n-3/20:1n-7)	
**Pheophytin**		
*Saccharina japonica*	**MGDG, DGDG, SQDG:** (C14:0, C16:0, C16:1, C18:0, C18; 1n-9, C18:2n-6, C18:3n-3, C18:4n-3, C20:4n-6, C20:5n-3)	Lee et al. (2004)
*Sargassum vulgare*	**MGDG:** (C16:0/C19:1), **DGDG:** (C16:0/C16:1), and **SQDG:** (C19:0/C16:0)	Plouguerné et al. (2020)
*Cladosiphon okamuranus*	**MGDG:** (18:3n-3/16:3n-3)	Terasaki and Itabashi (2003)
*Sargassum thunbergii*	**MGDG: 20:5/18:4, 18:3/18:4**	Kim et al. (2007)
*Saccharina cichorioides*	**MGDG, DGDG, SQDG:** (C16:0, C16:1n-7, C18:1n-9, C18:3n-6, C18:4n-3, C20:5n-3)	Logvinov et al. (2015)
*Sargassum pallidum*	**MGDG, DGDG, SQDG:** (C16:0, C16:1n-7, C18:1n-9, C18:2n-6, C18:3n-3, C18:4n-3, C20:4n-6)	Logvinov et al. (2015)
*Fucus spiralis*	**MGDG:** C20:5/C18:1, C20:5/C18:3	Lopes et al. (2014)
*Fucus vesiculosus*	**MGDG:** (18:1/14:0, 16:1/16:0; 18:4/16:0, 18:3/16:1; 18:3/16:0; 18:2/16:0, 18:1/16:1; 18:1/16:018:4/18:4; 18:3/18:4; 18:3/18:3; 18:2/18:3, 20:5/16:0; 20:4/16:0, 18:2/18:2; 20:5/18:4; 20:5/18:3, 20:4/18:4; 20:5/18:2, 20:4/18:3; 20:5/18:1, 20:4/18:2; 20:4/18:1; 20:4/18:0; 20:5/20:4; 20:4/20:4)	da Costa et al. (2019)
	**DGDG:** (14:0/18:2; 14:0/18:1, 16:0/16:1; 18:3/16:0; 18:2/16:0, 18:1/16:1; 18:1/16:0; 18:0/16:0; 18:3/18:4; 18:3/18:3; 20:5/16:0, 18:2/18:3; 20:4/16:0, 18:2/18:2; 18:1/18:2; 18:1/18:1; 18:1/18:0; 20:5/18:4; 20:5/18:3, 20:4/18:4; 20:4/18:3, 20:5/18:2; 20:4/18:2, 20:5/18:1; 20:4/18:1; 20:4/18:0)	
	**SQDG:** 14:0/14:0; 16:0/14:1; 16:0/14:0; 18:3/14:0; 18:2/14:0; 18:1/14:0; 18:0/14:0; 18:3/16:1; 18:3/16:0; 18:2/16:0; 18:1/16:0; 20:5/16:0; 20:4/16:0	
**Rhodophyta**		
*Solieria chordalis*	**MGDG:** (14:0/16:1)	Kendel et al. (2015)
*Exophyllum wentii*	MGDG (20:4n-6/16:0)	Honda et al. (2016)
	DGDG (20:4n-6/16:0)	
	SQDG (16:0 and 20:4n-6 acids)	
*Gracilaria vermiculophylla*	**MGDG:**C20:4n-6/C20:4n-6, C20:4n-6/C16:0	Muhammad et al. (2008)
	**DGDG:**C20:4n-6/C16:0, C20:4n-6/C16:0	
	**SQDG:** C20:4n-6/C16:0, C14:0/C16:0, C20:4n-6/C16:0	
*Tichocarpus crinitus*	**MGDG, DGDG, SQDG:** C16:0, C18:1n-9, C20:5n-3	Logvinov et al. (2015)
*Chondria armata*	**MGDG:**C20:5/C16:0	Al-Fadhli et al. (2006)
	**SQDG:** (C16:0/C16:0)	

### 2.3 Pharmacological effects and mechanisms of glyceroglycolipids

Unlike polysaccharides and fatty acids in marine algae, the therapeutic benefits of glyceroglycolipids remain poorly understood in the current literature because of their limited natural abundance and absorption. Nevertheless, glyceroglycolipids can be digested and absorbed by the gastrointestinal tract (Bajwa and Sastry, 1974). Andersson et al. (1995) showed that pancreatic lipase-related proteins could hydrolyze galactosylglycerides into galactosylmonoglycerides (MGMGs) and diglycerides (DGMGs), then into galactosylglycerol (MGG) and diglycerides (DGG), and finally into galactosylglycerol and glycerol. Andersson et al. (1996) also reported that although the hydrolysis and cleavage of thioisorhamnosylglycerol- and galactosylglycerol-based skeleton structure might not occur in the colon, they could be further hydrolyzed into sugar and glycerin in the cecum, indicating that glyceroglycolipids are absorbed and transformed at different degrees in the intestine and that their biological activity could be determined by the absorption and transformation of their glycosyl structure and unsaturated fatty acid *in vivo*. Hence, this review investigates the pharmacology of different structural types of glyceroglycolipids in marine algae by mainly focusing on their anti-oxidant and anti-inflammatory properties, anti-viral activity, and anti-tumor immunity.

#### 2.3 1 Anti-oxidant activities

Excessive oxygen free radicals have been associated with cardiovascular disease, aging, skin damage, and cancer (Block et al., 1983; Hassan and Abdel-Aziz, 2010). Cells and tissues have a complex anti-oxidant system to selectively scavenge excess free radicals and avoid damage to cellular structures. Therefore, supplementing exogenous anti-oxidants into diet and plant derivatives might have potential health benefits. In particular, although it is known that glycerolipids from marine algae have significant anti-oxidant activities, these roles have not been fully explored.

Genetic evidence suggests that membranes rich in polyunsaturated fatty acids (PUFAs) act as supramolecular anti-oxidants that capture reactive oxygen species, thereby limiting damage to proteins. This process generates lipid-fragmentation products, including malondialdehyde (MDA), an archetypal marker of PUFA oxidation. MGDG accelerates the rate at which MDA is metabolized (Schmid-Siegert et al., 2016). Another study showed that SQDG significantly inhibited LPS-induced NO production in RAW264.7 cells without affecting cell viability (Gao et al., 2014). Scavenging free radicals were shown to play important roles in human health, and marine algae-derived glyceroglycolipids were shown to possess significant anti-oxidant activities.

Overall, we found that the anti-oxidant mechanism of glyceroglycolipids could be explained by the fact that its unsaturated fatty acids play an important role in metabolites produced by lysis in the body, inhibiting the production of oxidizing substances such as NO in cells. As a result, the study of unsaturated fatty acid chains of glyceroglycolipids might become a hot topic in the future.

#### 2.3.2 Anti-inflammatory activities

Glyceroglycolipids of natural origins have immunomodulatory properties (Cheng et al., 2016). Marine algae-derived glyceroglycolipids are immunosuppressive and can reduce inflammation by activating cells with immunosuppressive functions, such as regulatory T cells (Treg) (Mueller et al., 2010; Du et al., 2015). MGDG, DGDG, and SQDG have been found to exert anti-inflammatory effects by inducing superoxide anion production in leukocytes and chemotaxis of human peripheral neutrophils to inhibit croton oil-induced ear edema in mice (Bruno et al., 2005). In addition, MGDG and DGDG showed significant activity in lipopolysaccharide-stimulated human THP-1 macrophages to inhibit TNF-α production and activate anti-inflammatory mechanisms in the organism (De Los Reyes et al., 2016). Another study demonstrated that MGDG activated the cyclooxygenase-2 (COX-2) pathway and had anti-inflammatory activities on human articular cartilage (Ulivi et al., 2011). Furthermore, glyceroglycolipids of marine algae extracts were found to inhibit TNF-α-induced IL-8 production in the HT-29 cell line (Kiem et al., 2012). These studies suggest that glyceroglycolipids from marine algae might have anti-inflammatory effects.

**FIGURE 3:**
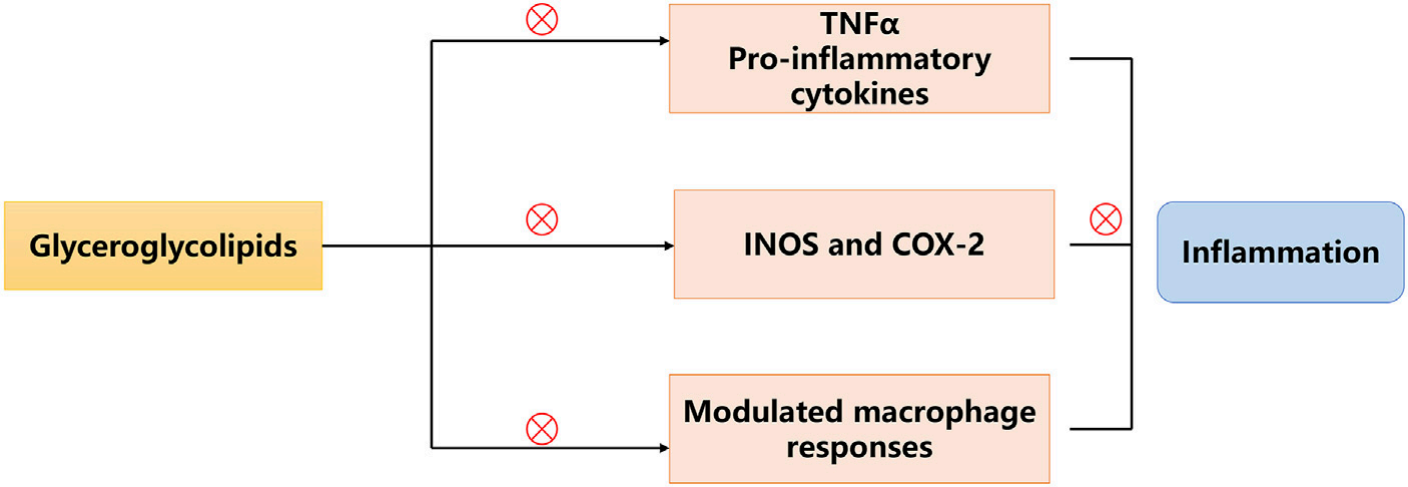
Anti-inflammatory mechanism of glyceroglycolipids.

Inflammatory mediators such as PGE2 and NO play key roles in every step of inflammation and have been implicated in the pathogenesis of various inflammatory diseases. Glycerol glycolipids were reported to inhibit LPS-induced protein and mRNA expression of iNOS and COX-2 in RAW264.7 macrophages and strongly inhibit NO and PGE2 production. At the same time, it also regulated the response of macrophages, as shown in Figure 3.

#### 2.3.3 Anti-viral activities

Anti-viral chemotherapy is important in preventing and treating many important viral infections (De Clercq and Field, 2006; Plouguerné et al., 2013). However, the acceptance of anti-viral chemotherapy has been challenging mainly because clinicians were made to believe that viral inhibitors can have toxic effects on the host (Hayashi et al., 2019; Cénat et al., 2020; Sun et al., 2020). Currently, novel sources of anti-viral drugs are being actively researched. In the last few years, marine algae compounds have been examined for potential use in anti-viral drugs, of which glyceroglycolipids represent an emerging anti-viral secondary metabolite (Zhang X. et al., 2020). MGDG and DGDG are resistant to herpes simplex virus 2 (HSV-2) and can make the virus lose its ability to bind to cells and inhibit its replication *in vivo* (Hayashi et al., 2019; Wang et al., 2020). In addition, MGDG has anti-viral activity and can also combine with other substances to form immune complexes, which can stimulate the production of specific antibodies *in vivo* to inhibit influenza virus hemagglutinin (HA), thereby demonstrating its anti-viral effects (Nina et al., 2017). One study found that isolating SQDG from brown algae of Brazil also had inhibitory effects on herpes virus (HSV-1 and HSV-2) (Nina et al., 2017). In addition, a previous study reported that SQDG could inhibit HIV replication with the same effects as HIV reverse transcriptase through cell experiments (Gustafson et al., 1989). Ash et al. (2017) also studied the anti-viral activity of SQDG and found that the anti-viral mechanism of SQDG was mainly through the combination with DNA to induce apoptosis.

**FIGURE 4:**
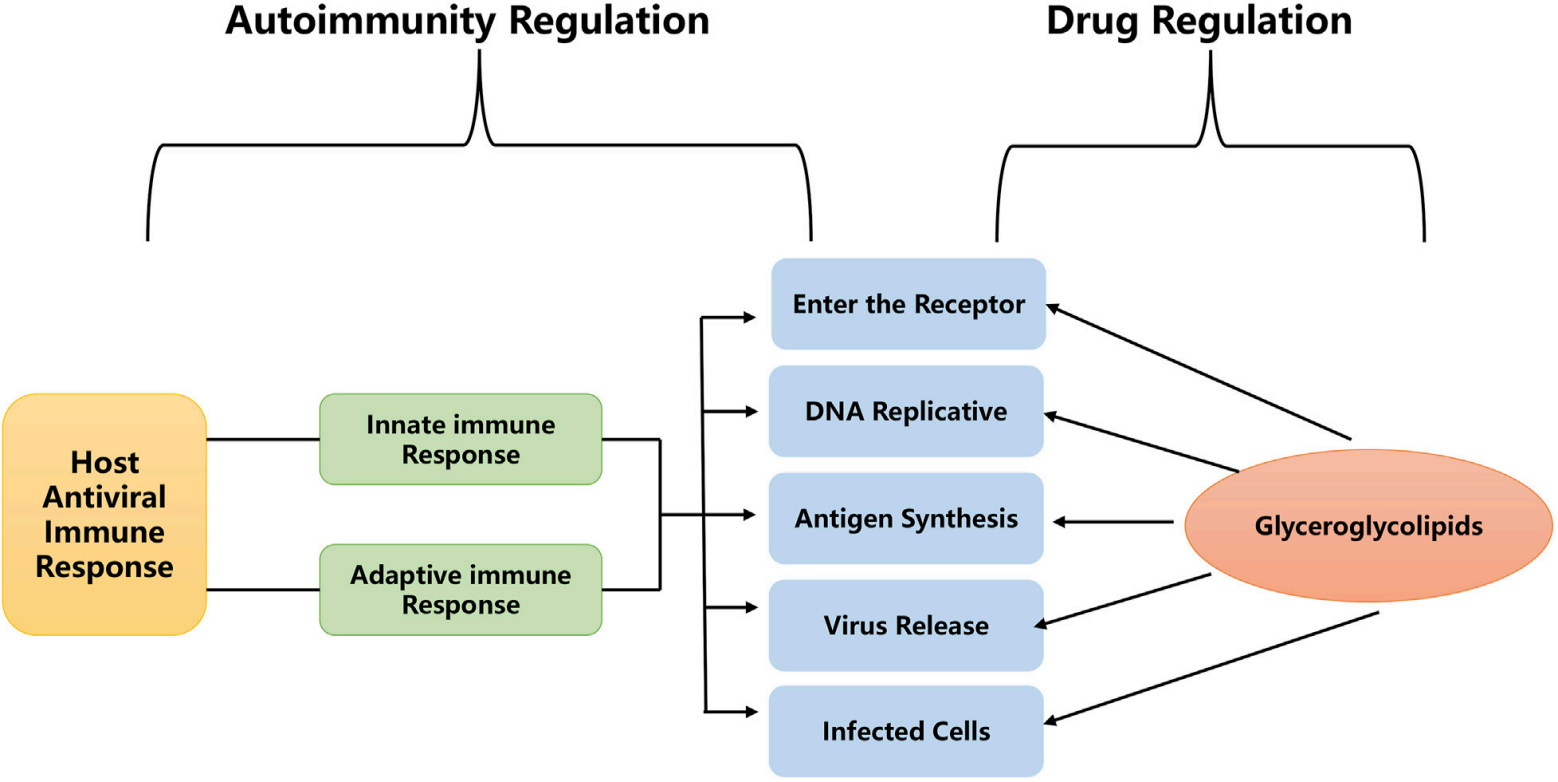
Potential anti-viral mechanism of glyceroglycolipids.

Based on the existing literature, we can conclude that there are three main anti-viral mechanisms of glycerol glycolipids: 1) preventing viral infection from entering the host cell by directly inhibiting the binding of the virus to the cell surface; 2) preventing the virus from exfoliating in the host cell by binding at the variable configuration site of the viral capsid; and 3) inhibiting the transcriptional replication process of the virus from the host cell by interfering with replication enzymes such as reverse transcriptase or preventing the formation of reverse transcriptase proteins of cellular messenger RNAs (Figure 4). Thus, marine algae glyceroglycolipids might be a primary candidate for the future anti-viral drug discovery work.

#### 2.3.4 Anti-bacterial activities

**FIGURE 5:**
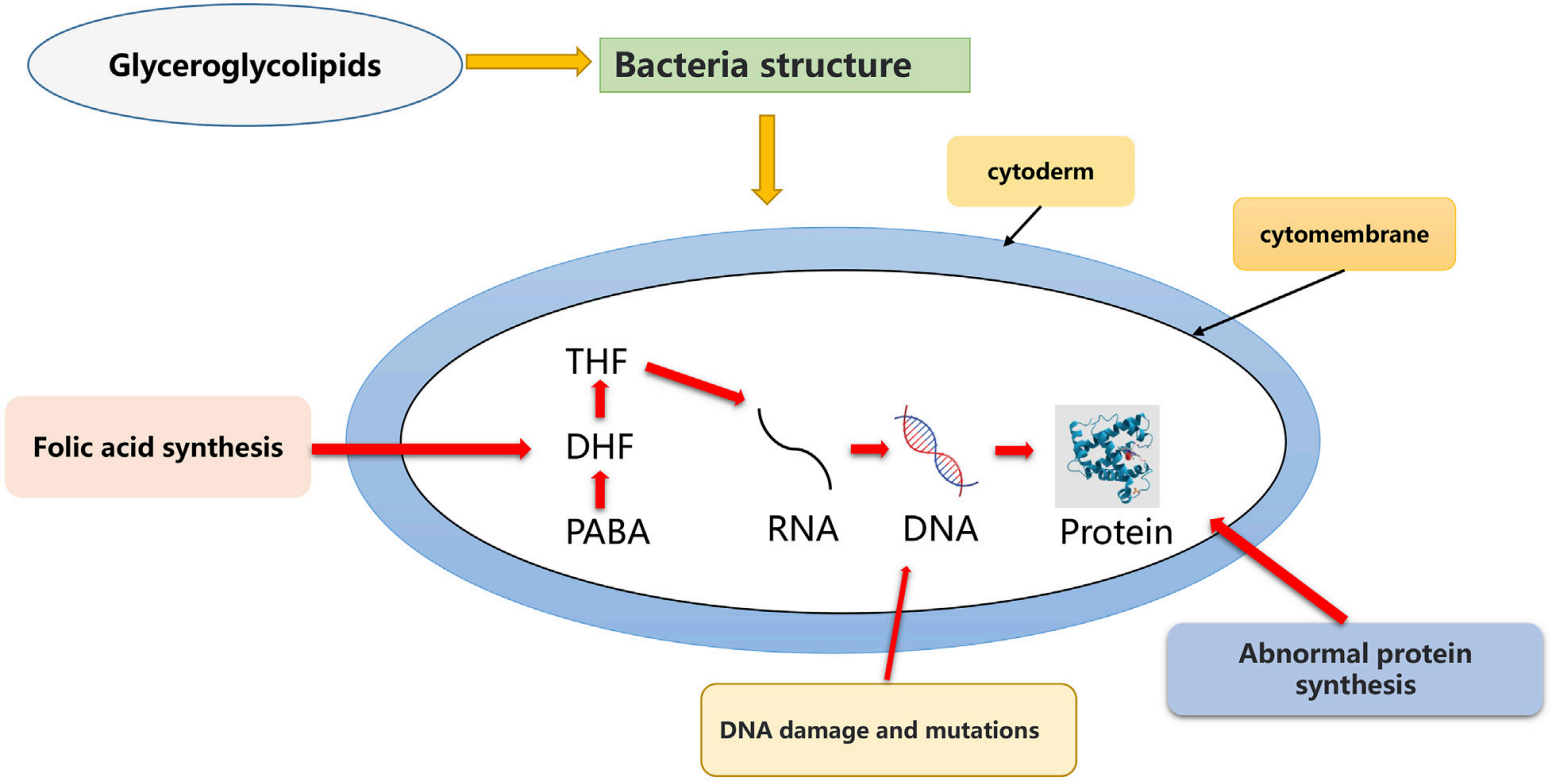
Potential mechanism of anti-bacterial activity of glyceroglycolipids.

Many highly effective antibiotics against bacteria and fungi are already in widespread use. However, due to the poor membrane permeability of many hydrophilic antibiotics, the choice of antimicrobial drugs for intracellular use is limited (Jiang et al., 2020). Recent studies have found glyceroglycolipids to have anti-bacterial activities. Dai et al. (2001) found that glycoglycerolipid could inactivate *Bacillus*. Plouguerné et al. (2014) reported that MGDG had strong anti-bacterial activity against *Haemophilus influenzae*, *Legionella pneumophila*, *Propionibacterium acnes*, *Streptococcus pyogenes*, *Clostridium difficile*, and *Staphylococcus aureus*. In addition, Parveez Ahamed et al. (2017) found that the acyl structure of glycerol glycolipid was related to its anti-bacterial activity. In addition, Furukawa et al. (2006) found that SQDG inhibited the proliferation of *E. coli* JM190 cells, while MGDG and DGDG did not show similar activities. These studies show the differences in the anti-bacterial activities of different structural types of glycoglycerolipids. However, there are few studies on the antimicrobial activity of glycolipids, of which most of the experiments have focused on their mechanism of action *in vitro*. Figure 5 summarizes the mechanism of action of various antimicrobial agents. Glyceroglycolipids were shown to disrupt mitochondrial functions in fungal cells in MTT assay (Lopes et al., 2013). Many dehydrogenases, such as lactate dehydrogenase and succinate, are involved in the mitochondrial respiratory chain. Glyceroglycolipids exert their anti-microbial effects by increasing the mitochondrial respiration rate and interaction with these enzymes, leading to higher ROS production.

#### 2.3.5 Anti-tumor activities

Cancer is a major disease that reduces life expectancy. Currently, the main methods to treat tumors are surgery, radiotherapy, chemotherapy, and hormone therapy (Zhang W. et al., 2020). Although these methods can have certain therapeutic effects, in many cases, they also have serious adverse events (Rath et al., 2019; Yan et al., 2019). Therefore, research on more effective oncology drugs with lesser toxicity is of great significance and has become a research hotspot. Numerous studies have attempted to explain the strong anti-tumor activities of glyceroglycolipids. Shirahashi et al. (1993) reported that galactoglyceride significantly inhibited the growth of tumors. One study showed that DGDG had inhibitory effects on dermal papilloma in mice (Tokuda et al., 1996). Colombo et al. (2005) found that galactosylglycerides with more branched and unsaturated acyl chains had strong inhibitory effects on mouse skin tumors and proved that the acyl structure of glycerosylglycerides had a great influence on the activity of the glyceroglycolipids. In addition, they also found that the presence of aliphatic or aromatic rings in glycerol glycolipids had a negative effect on their anti-tumor activity (Colombo et al., 2006) and revealed that galactosylglycerides played a therapeutic role in cancer prevention and treatment by inhibiting the growth of tumor cells and blocking the expression of protein kinase C (Colombo et al., 2011). In addition, Akasaka et al. (2013) found that MGDG, a DNA polymerase inhibitor, can enhance the anti-proliferation effects of gemcitabine on human pancreatic cancer cells. However, Maeda et al. (2005) reported that glycerol glycolipid was not only an inhibitor of DNA polymerase but also a growth inhibitor of human gastric cancer cells, and its activity was stronger when it was hydrolyzed by lipase. Maeda et al. (2009) also reported that glycerol glycolipid had an inhibitory effect on the growth of transplanted tumor sarcoma and colon tumors in mice. Dangate et al. (2009) found that SQDG had anti-tumor effects and inhibited tumor promoters. Ogunsina et al. (2013) suggested that the glycosyl structure of glycerols was related to the activity of anti-tumor glycerides, and the anti-tumor activity of galactosyls was greatly lost when they were replaced. Andrianasolo et al. (2008) found that galactoglyceride induced apoptosis at micromolecular concentration and played an important role in the anti-tumor mechanism of glyceroglycolipids.

In summary, the three functions of glyceroglycolipids are crucial for tumor invasiveness and occur through different mechanisms at different sites (Table 2). Many studies have reported that glyceroglycolipids have unique advantages in immune regulation and anti-tumor activity. The anti-tumor effects of glyceroglycolipids are an active focus of research in pharmacology.

**TABLE 2:** Anti-tumor activities and mechanism of glyceroglycolipids.

Structure type	Anti-tumor activities	Mechanism	Ref
MGDG	Breast cancer	Inhibition of DNA polymerases and growth inhibition of tumor cells	Akasaka et al. (2013)
MGDG	Prostatic cancer	Inhibition of DNA polymerases and growth inhibition of tumor cells	Akasaka et al. (2013)
MGDG	Lymphocytic leukemia	Inhibition of DNA polymerases and growth inhibition of tumor cells	Akasaka et al. (2013)
MGDG	Tumors of epithelial origin	Induce apoptosis upstream of Bax and Bak	Andrianasolo et al. (2008)
DGDG	Skin papilloma	Decrease protein kinase C protein translocation to membranes	Colombo et al. (2005)
DGDG	Stomach adenocarcinoma	Inhibition of DNA polymerases and growth inhibition of tumor cells	Ramm et al. (2004)
DGDG	Hepatocellular carcinoma	Inhibition of DNA polymerases and growth inhibition of tumor cells	Ramm et al. (2004)
SQDG	Skin papilloma	Inhibiting the Epstein–Barr virus activation	Dangate et al. (2009)
MGDG and DGDG	Melanoma	Inhibition of DNA polymerases and growth inhibition of tumor cells	Maeda et al. (2005)
MGDG and DGDG	Pancreatic cancer	Inhibition of DNA polymerases and growth inhibition of tumor cells; cytotoxicity	Maeda et al. (2005); Akasaka et al. (2016)
MGDG and DGDG	Lung adenocarcinoma	Moderately cytotoxic toward tumor cell	Perez Gutierrez and Lule, (2005)
MGDG and DGDG	Human oral epidermoid carcinoma	Moderately cytotoxic toward tumor cell	Perez Gutierrez and Lule, (2005)
MGDG, DGDG, and SQDG	Colon tumor	The protein expression level of proliferating cell nuclear antigen (PCNA) was decreased	Maeda et al. (2009)

#### 2.3.6 Treatment of metabolic diseases

**FIGURE 6:**
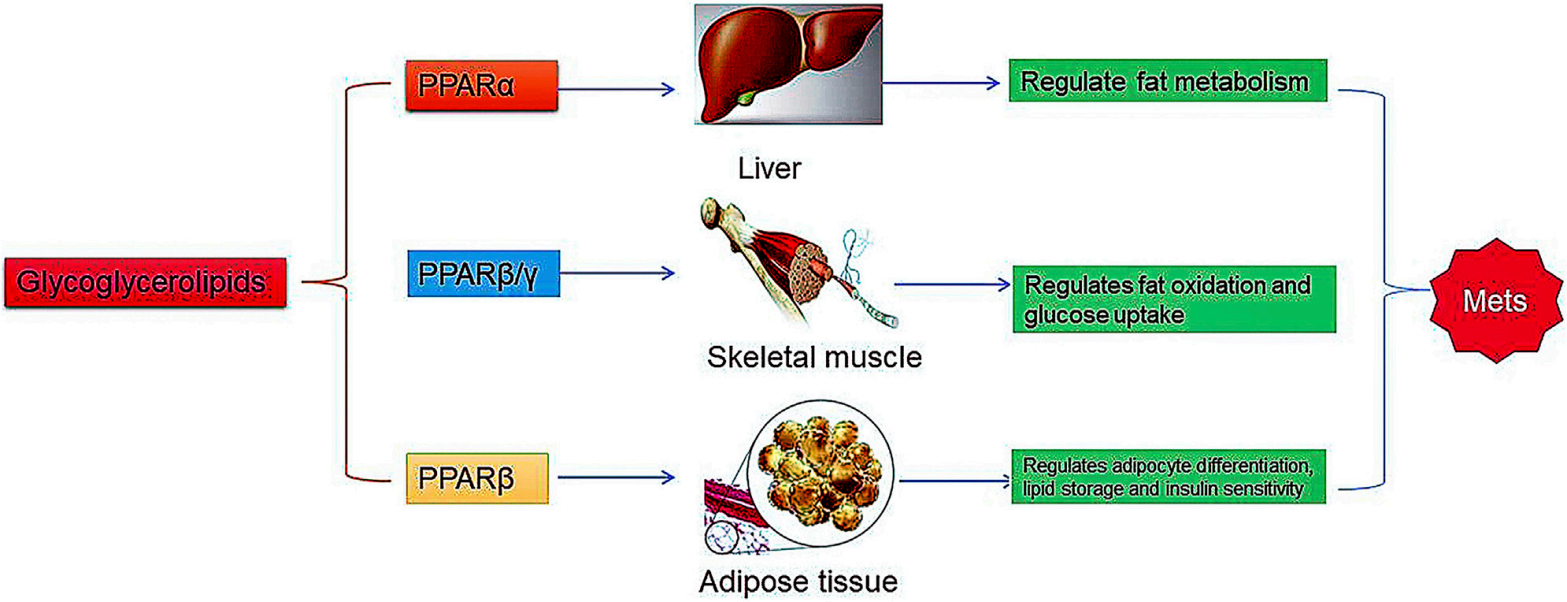
Effects of glyceroglycolipids on PPARs.

Obesity and obesity-initiated metabolic syndrome are characterized by high levels of cholesterol and lipids in blood and intracellular fat accumulation in adipose tissues. Some researchers showed that glyceroglycolipids significantly reduced adipogenic PPAR-γ protein expression in differentiated 3T3-L1 cells (Chen et al., 2022; Prabhakar et al., 2022). Peroxisome proliferator-activated receptors (PPARs) are a family of ligand-regulated nuclear receptors that include PPAR-α, PPAR-β/δ, and PPAR-γ (Lee et al., 2022). These receptors play a significant role in the regulation of transcription, energy and lipid metabolism, and thermogenesis (Lee et al., 2020). One study identified a specific type of MGDG in which the acyl groups acted as a potent PPAR-γ (peroxisome proliferator-activated receptor γ) ligand (Figure 6) (Pinto et al., 2021). These studies suggest that algal-derived glycerolipids might be considered potential activation targets of the PPAR family, indicating the need for further in-depth analysis of marine algae and their natural products.

## 4 Conclusion

Glyceroglycolipids represent a broad class of biologically active natural products with a wide variety of molecular structures and biological functions, many of which are essential for life. They are naturally found in marine algae and appear to possess pharmacological activities such as anti-cancer, anti-bacterial, anti-viral, and anti-oxidant properties. There are various mechanisms underlying the activities of glyceroglycolipids, including the combination of cell signaling pathways at various stages of diseases.

In terms of anti-oxidants, the polyunsaturated fatty acids in glycerol glycolipids can reduce free peroxidation and rapidly scavenge malondialdehyde and are considered the main mechanisms of action. Meanwhile, glycerol glycolipids inhibited LPS-induced protein and mRNA expression of iNOS and COX-2 in RAW264.7 macrophages and strongly inhibited NO and PGE2 production. This mechanism indicates that glycerol glycolipids possess strong anti-inflammatory activities. In addition, glycerol glycolipids are also highly active against pathogenic microorganisms. Glyceroglycolipids prevent viral infection from entering the host cell by directly inhibiting the binding of the virus to the cell surface membrane. At the same time, polyunsaturated fatty acids bind to the variable conformation site of the viral shell and prevent viral shedding in host cells. Glycerolipids also exert anti-viral effects by interfering with replication enzymes (e.g., reverse transcriptase) or by preventing the formation of reverse transcriptase proteins of cellular messenger RNA, which inhibit the transcriptional replication process of viruses from host cells. In addition, the interaction of polyunsaturated fatty acids in glyceroglycolipids with various dehydrogenases increases the mitochondrial respiration rate, leading to higher ROS production, thus promoting anti-microbial effects. Glyceroglycolipids also have anti-cancer activities. Its main mechanisms of action include the inhibition of DNA polymerase and inhibition of tumor cell growth by inducing apoptosis upstream of Bax and Bak, reducing the transfer of protein kinase C protein to the membrane. The biological activity is closely related to the glycosyl and acyl chains of glyceroglycolipids. Consequently, it could be used in drugs for treating various diseases. However, due to the low natural abundance and difficult separation, research on the application of glycerolipids is limited. Meanwhile, the current research on glyceroglycolipids in marine algae remains under-investigated, urging the need for greater consideration due to their promising benefits.

## References

[B1] Agatonovic-Kustrin S. Kustrin E. Angove M. J. Morton D. W. (2018). A screening method for cardiovascular active compounds in marine algae. J. Chromatogr. A 1550, 57–62. 10.1016/j.chroma.2018.03.054 29615323

[B2] Akasaka H. Mizushina Y. Yoshida K. Ejima Y. Mukumoto N. Wang T. (2016). MGDG extracted from spinach enhances the cytotoxicity of radiation in pancreatic cancer cells. Radiat. Oncol. 11 (1), 153. 10.1186/s13014-016-0729-0 27876069 PMC5120455

[B3] Akasaka H. Sasaki R. Yoshida K. Takayama I. Yamaguchi T. Yoshida H. (2013). Monogalactosyl diacylglycerol, a replicative DNA polymerase inhibitor, from spinach enhances the anti-cell proliferation effect of gemcitabine in human pancreatic cancer cells. Biochim. Biophys. Acta 1830 (3), 2517–2525. 10.1016/j.bbagen.2012.11.004 23174220

[B4] Al-Fadhli A. Wahidulla S. Souza L. (2006). Glycolipids from the red alga chondria armata (kutz.) okamura. Glycobiology 16 (10), 902–915. 10.1093/glycob/cwl018 16799167

[B5] Alves E. Dias M. Lopes D. Almeida A. Domingues M. Rey F. (2020). Antimicrobial lipids from plants and marine organisms: An overview of the current state-of-the-art and future prospects. Antibiot. (Basel, Switz. 9 (8), 441. 10.3390/antibiotics9080441 PMC7459900 32722192

[B6] Andersson L. Bratt C. Arnoldsson K. C. Herslöf B. Olsson N. U. Sternby B. (1995). Hydrolysis of galactolipids by human pancreatic lipolytic enzymes and duodenal contents. J. Lipid Res. 36 (6), 1392–1400. 10.1016/S0022-2275(20)41146-0 7666015

[B7] Andersson L. Carrière F. Lowe M. E. Nilsson K. Verger R. Nilsson A. (1996). Pancreatic lipase-related protein 2 but not classical pancreatic lipase hydrolyzes galactolipids. Biochim. Biophys. Acta 1302 (3), 236–240. 10.1016/0005-2760(96)00068-9 8765145

[B8] Andrianasolo E. H. Haramaty L. Vardi A. White E. Lutz R. Falkowski P. (2008). Apoptosis-inducing galactolipids from a cultured marine diatom, Phaeodactylum tricornutum. J. Nat. Prod. 71 (7), 1197–1201. 10.1021/np800124k 18570469 PMC2866075

[B9] Ash A. Bharitkar Y. P. Murmu S. Hazra A. Ravichandiran V. Kar P. K. (2017). Ultrastructural changes in raillietina (Platyhelminthes: Cestoda), exposed to sulfonoquinovosyldiacylglyceride (SQDG), isolated from neem (Azadirachta indica). Nat. Prod. Res. 31 (20), 2445–2449. 10.1080/14786419.2017.1305383 28347172

[B10] Bajwa S. S. Sastry P. S. (1974). Degradation of monogalactosyl diglyceride and digalactosyl diglyceride by sheep pancreatic enzymes. Biochem. J. 144 (2), 177–187. 10.1042/bj1440177 4462578 PMC1168484

[B11] Bharadwaj K. K. Ahmad I. Pati S. Ghosh A. Sarkar T. Rabha B. (2022). Potent bioactive compounds from seaweed waste to combat cancer through bioinformatics investigation. Front. Nutr. 9, 889276. 10.3389/fnut.2022.889276 35529456 PMC9075044

[B12] Block M. A. Dorne A. J. Joyard J. Douce R. (1983). Preparation and characterization of membrane fractions enriched in outer and inner envelope membranes from spinach chloroplasts. II. Biochemical characterization. J. Biol. Chem. 258 (21), 13281–13286. 10.1016/S0021-9258(17)44113-5 6630230

[B13] Bruno A. Rossi C. Marcolongo G. Di Lena A. Venzo A. Berrie C. P. (2005). Selective *in vivo* anti-inflammatory action of the galactolipid monogalactosyldiacylglycerol. Eur. J. Pharmacol. 524 (1-3), 159–168. 10.1016/j.ejphar.2005.09.023 16253232

[B14] Cénat J. M. Mukunzi J. N. Noorishad P. G. Rousseau C. Derivois D. Bukaka J. (2020). A systematic review of mental health programs among populations affected by the Ebola virus disease. J. Psychosom. Res. 131, 109966. 10.1016/j.jpsychores.2020.109966 32087433

[B15] Cepas V. Gutiérrez-Del-Río I. López Y. Redondo-Blanco S. Gabasa Y. Iglesias M. J. (2021). Microalgae and cyanobacteria strains as producers of lipids with antibacterial and antibiofilm activity. Mar. Drugs 19 (12), 675. 10.3390/md19120675 34940674 PMC8709229

[B16] Chen B. Chen H. Qu H. Qiao K. Xu M. Wu J. (2022). Photoprotective effects of Sargassum thunbergii on ultraviolet B-induced mouse L929 fibroblasts and zebrafish. BMC Complement. Med. Ther. 22 (1), 144. 10.1186/s12906-022-03609-x 35597942 PMC9123674

[B17] Chen H. Wang Q. (2021). Regulatory mechanisms of lipid biosynthesis in microalgae. Biol. Rev. Camb. Philos. Soc. 96 (5), 2373–2391. 10.1111/brv.12759 34101323

[B18] Cheng J. J. Chao C. H. Chang P. C. Lu M. K. (2016). Studies onanti-inflammatory activity of sulfated polysaccharides from cultivated fungi antrodia cinnamomea. Food Hydrocoll. 53, 37–45. 10.1016/j.foodhyd.2014.09.035

[B19] Colombo D. Franchini L. Toma L. Ronchetti F. Nakabe N. Konoshima T. (2005). Anti-tumor-promoting activity of simple models of galactoglycerolipids with branched and unsaturated acyl chains. Eur. J. Med. Chem. 40 (1), 69–74. 10.1016/j.ejmech.2004.09.021 15642411

[B20] Colombo D. Franchini L. Toma L. Ronchetti F. Tanaka R. Takayasu J. (2006). Cyclic and branched acyl chain galactoglycerolipids and their effect on anti-tumor-promoting activity. Eur. J. Med. Chem. 41 (12), 1456–1463. 10.1016/j.ejmech.2006.07.007 16996658

[B21] Colombo D. Tringali C. Franchini L. Cirillo F. Venerando B. (2011). Glycoglycerolipid analogues inhibit PKC translocation to the plasma membrane and downstream signaling pathways in PMA-treated fibroblasts and human glioblastoma cells, U87MG. Eur. J. Med. Chem. 46 (5), 1827–1834. 10.1016/j.ejmech.2011.02.043 21388717

[B22] da Costa E. Domingues P. Melo T. Coelho E. Pereira R. Calado R. (2019). Lipidomic signatures reveal seasonal shifts on the relative abundance of high-valued lipids from the Brown algae fucus vesiculosus. Mar. Drugs 17 (6), 335. 10.3390/md17060335 PMC6627367 31167455

[B23] Dai J. Q. Zhu Q. X. Zhao C. Y. Yang W. Li Y. (2001). Glyceroglycolipids from serratula strangulata. Phytochemistry 58 (8), 1305–1309. 10.1016/s0031-9422(01)00308-9 11738426

[B24] Dangate M. Franchini L. Ronchetti F. Arai T. Iida A. Tokuda H. (2009). 2-O-beta-d-glucopyranosyl-sn-glycerol based analogues of sulfoquinovosyldiacylglycerols (SQDG) and their role in inhibiting Epstein-Barr virus early antigen activation. Bioorg. Med. Chem. 17 (16), 5968–5973. 10.1016/j.bmc.2009.06.064 19631552

[B25] De Clercq E. Field H. J. (2006). Antiviral prodrugs the development of successful prodrug strategies for antiviral chemotherapy. Br. J. Pharmacol. 147 (1), 1–11. 10.1038/sj.bjp.0706446 16284630 PMC1615839

[B26] de Los Reyes C. Ortega M. J. Rodríguez-Luna A. Talero E. Motilva V. Zubía E. (2016). Molecular characterization and anti-inflammatory activity of galactosylglycerides and galactosylceramides from the microalga isochrysis galbana. J. Agric. Food Chem. 64 (46), 8783–8794. 10.1021/acs.jafc.6b03931 27786470

[B27] Demé B. Cataye C. Block M. A. Maréchal E. Jouhet J. (2014). Contribution of galactoglycerolipids to the 3-dimensional architecture of thylakoids. FASEB J. 28 (8), 3373–3383. 10.1096/fj.13-247395 24736411

[B28] Domingues M. R. Calado R. (2022). Lipids of marine algae-biomolecules with high nutritional value and important bioactive properties. Biomolecules 12 (1), 134. 10.3390/biom12010134 35053282 PMC8774186

[B29] Du B. Lin C. Bian Z. Xu B. (2015). An insight into anti-inflammatory effects of fungal beta-glucans. Trends Food Sci. Technol. 41 (1), 49–59. 10.1016/j.tifs.2014.09.002

[B30] Furukawa T. Nishida M. Hada T. Kuramochi K. Sugawara F. Kobayashi S. (2006). Inhibitory effect of sulfoquinovosyl diacylglycerol on prokaryotic DNA polymerase I activity and cell growth of *Escherichia coli* . J. Oleo Sci. 56 (1), 43–47. 10.5650/jos.56.43 17693698

[B31] Gao Q. M. Yu K. Xia Y. Shine M. B. Wang C. Navarre D. R. (2014). Mono-and digalactosyldiacylglycerol lipids function nonredundantly to regulate systemic acquired resistance in plants. Cell Rep. 9 (5), 1681–1691. 10.1016/j.celrep.2014.10.069 25466253

[B32] Guiry M. D. Guiry G. M. de maio de (2021). AlgaeBase. Galway: World-wide electronic publication, National University of Ireland.

[B33] Guschina I. A. Hayes A. J. Ormerod S. J. (2020). Polystyrene microplastics decrease accumulation of essential fatty acids in common freshwater algae. Environ. Pollut. 263, 114425. 10.1016/j.envpol.2020.114425 32229374

[B34] Gustafson K. R. Cardellina J. H. 2nd Fuller R. W. Weislow O. S. Kiser R. F. Snader K. M. (1989). AIDS-antiviral sulfolipids from cyanobacteria (blue-green algae). J. Natl. Cancer Inst. 81 (16), 1254–1258. 10.1093/jnci/81.16.1254 2502635

[B35] Hassan H. A. Abdel-Aziz A. F. (2010). Evaluation of free radical-scavenging and anti-oxidant properties of black berry against fluoride toxicity in rats. Food Chem. Toxicol. 48 (8-9), 1999–2004. 10.1016/j.fct.2010.05.018 20472017

[B36] Hayashi K. Lee J. B. Atsumi K. Kanazashi M. Shibayama T. Okamoto K. (2019). *In vitro* and *in vivo* anti-herpes simplex virus activity of monogalactosyl diacylglyceride from coccomyxa sp. kj (ipod ferm bp-22254), a green microalga. PLoS ONE 14 (7), e0219305. 10.1371/journal.pone.0219305 31310628 PMC6634382

[B37] Heinz E. Roughan P. G. (1983). Similarities and differences in lipid metabolism of chloroplasts isolated from 18:3 and 16:3 plants. Plant Physiol. 72 (2), 273–279. 10.1104/pp.72.2.273 16662992 PMC1066223

[B38] Hölzl G. Dörmann P. (2007). Structure and function of glycoglycerolipids in plants and bacteria. Prog. Lipid Res. 46 (5), 225–243. 10.1016/j.plipres.2007.05.001 17599463

[B39] Honda M. Ishimaru T. Itabashi Y. (2016). Lipid classes, fatty acid composition, and glycerolipid molecular species of the red alga gracilaria vermiculophylla, a prostaglandin-producing seaweed. J. Oleo Sci. 65 (9), 723–732. 10.5650/jos.ess16026 27581490

[B40] Hoyo J. Guaus E. Torrent-Burgués J. (2016). Monogalactosyldiacylglycerol and digalactosyldiacylglycerol role, physical states, applications and biomimetic monolayer films. Eur. Phys. J. E Soft Matter 39 (3), 39. 10.1140/epje/i2016-16039-0 27021656

[B41] Jiang Y. Han M. Bo Y. Feng Y. Li W. Wu J. R. (2020). "Metaphilic" cell-penetrating polypeptide-vancomycin conjugate efficiently eradicates intracellular bacteria via a dual mechanism. ACS Cent. Sci. 6 (12), 2267–2276. 10.1021/acscentsci.0c00893 33376787 PMC7760462

[B42] Kendel M. Wielgosz-Collin G. Bertrand S. Roussakis C. Bourgougnon N. Bedoux G. (2015). Lipid composition, fatty acids and sterols in the seaweeds ulva armoricana, and solieria chordalis from brittany (France): An analysis from nutritional, chemotaxonomic, and antiproliferative activity perspectives. Mar. Drugs 13 (9), 5606–5628. 10.3390/md13095606 26404323 PMC4584343

[B43] Kiem P. V. Minh C. V. Nhiem N. X. Cuong N. X. Tai B. H. Quang T. H. (2012). Inhibitory effect on TNF-α-induced IL-8 secretion in HT-29 cell line by glyceroglycolipids from the leaves of Ficus microcarpa. Arch. Pharm. Res. 35 (12), 2135–2142. 10.1007/s12272-012-1210-8 23263807

[B44] Kim Y. H. Kim E. H. Lee C. Kim M. H. Rho J. R. (2007). Two new monogalactosyl diacylglycerols from Brown alga Sargassum thunbergii. Lipids 42 (4), 395–399. 10.1007/s11745-007-3035-7 17406933

[B45] Lee H. G. Jayawardena T. U. Song K. M. Choi Y. S. Jeon Y. J. Kang M. C. (2022). Dietary fucoidan from a Brown marine algae (Ecklonia cava) attenuates lipid accumulation in differentiated 3T3-L1 cells and alleviates high-fat diet-induced obesity in mice. Food Chem. Toxicol. 162, 112862. 10.1016/j.fct.2022.112862 35157925

[B46] Lee H. G. Lu Y. A. Li X. Hyun J. M. Kim H. S. Lee J. J. (2020). Anti-obesity effects of grateloupia elliptica, a red seaweed, in mice with high-fat diet-induced obesity via suppression of adipogenic factors in white adipose tissue and increased thermogenic factors in Brown adipose tissue. Nutrients 12 (2), 308. 10.3390/nu12020308 PMC7071330 31991562

[B47] Lee I. A. Popov A. M. Sanina N. M. Kostetsky E. Y. Shnyrov V. L. Reunov A. V. (2004). Morphological and immunological characterization of immunostimulatory complexes based on glycoglycerolipids from laminaria japonica. Acta Biochim. Pol. 51 (1), 263–272. 10.18388/abp.2004_3619 15094848

[B48] Logvinov S. Gerasimenko N. Esipov A. Denisenko V. A. (2015). Examination of the structures of several glycerolipids from marine macroalgae by NMR and GC-MS. J. Phycol. 51 (6), 1066–1074. 10.1111/jpy.12338 26987002

[B49] Lopes G. Daletos G. Proksch P. Andrade P. B. Valentão P. (2014). Anti-inflammatory potential of monogalactosyl diacylglycerols and a monoacylglycerol from the edible Brown seaweed Fucus spiralis Linnaeus. Mar. Drugs 12 (3), 1406–1418. 10.3390/md12031406 24619274 PMC3967218

[B50] Lopes G. Pinto E. Andrade P. B. Valentao P. (2013). Antifungal activity of phlorotannins against dermatophytes and yeasts: Approaches to the mechanism of action and influence on Candida albicans virulence factor. PLoS One 8 (8), e72203. 10.1371/journal.pone.0072203 23951297 PMC3741211

[B51] Lordan S. Ross R. P. Stanton C. (2011). Marine bioactives as functional food ingredients: Potential to reduce the incidence of chronic diseases. Mar. Drugs 9 (6), 1056–1100. 10.3390/md9061056 21747748 PMC3131561

[B52] Loya S. Reshef V. Mizrachi E. Silberstein C. Rachamim Y. Carmeli S. (1998). The inhibition of the reverse transcriptase of HIV-1 by the natural sulfoglycolipids from cyanobacteria: Contribution of different moieties to their high potency. J. Nat. Prod. 61 (7), 891–895. 10.1021/np970585j 9677270

[B53] Maeda N. Hada T. Murakami-Nakai C. Kuriyama I. Ichikawa H. Fukumori Y. (2005). Effects of DNA polymerase inhibitory and anti-tumor activities of lipase-hydrolyzed glycolipid fractions from spinach. J. Nutr. Biochem. 16 (2), 121–128. 10.1016/j.jnutbio.2004.08.005 15681172

[B54] Maeda N. Kokai Y. Ohtani S. Hada T. Yoshida H. Mizushina Y. (2009). Inhibitory effects of preventive and curative orally administered spinach glycoglycerolipid fraction on the tumor growth of sarcoma and colon in mouse graft models. Food Chem. 112 (1), 205–210. 10.1016/j.foodchem.2008.05.059

[B55] Mancini I. Guella G. Defant A. Candenas M. Armesto C. Depentori D. (1998). Polar metabolites of the tropical green SeaweedCaulerpa taxifolia Which Is Spreading in the mediterranean sea: Glycoglycerolipids and stable enols (α\=Keto esters). Helv. Chim. Acta 81 (9), 1681–1691. 10.1002/(sici)1522-2675(19980909)81:9<1681::aid-hlca1681>3.0.co;2-4

[B56] Mandalka A. Cavalcanti M. Harb T. B. Toyota Fujii M. Eisner P. Schweiggert- (2022). Nutritional composition of beach-cast marine algae from the Brazilian coast: Added value for algal biomass considered as waste. Foods (Basel, Switz. 11(9), 1201. 10.3390/foods11091201 PMC9099717 35563924

[B57] Marcolongo G. de Appolonia F. Venzo A. Berrie C. P. Carofiglio T. Ceschi Berrini C. (2006). Diacylglycerolipids isolated from a thermophile cyanobacterium from the Euganean hot springs. Nat. Prod. Res. 20 (8), 766–774. 10.1080/14786410500176393 16753911

[B58] Michaud M. Prinz W. A. Jouhet J. (2017). Glycerolipid synthesis and lipid trafficking in plant mitochondria. FEBS J. 284 (3), 376–390. 10.1111/febs.13812 27406373 PMC6224293

[B59] Morimoto T. Nagatsu A. Murakami N. Sakakibara J. Tokuda H. Nishino H. (1995). Anti-tumour-promoting glyceroglycolipids from the green alga, Chlorella vulgaris. Phytochemistry 40 (5), 1433–1437. 10.1016/0031-9422(95)00458-j 8534400

[B60] Mueller M. Hobiger S. Jungbauer A. (2010). Anti-inflammatory activity of extracts from fruits, herbs and spices. Food Chem. 122 (4), 987–996. 10.1016/j.foodchem.2010.03.041

[B61] Muhammad I. I. Masaru T. Ryo N. Iijima N. Hara A. Fusetani N. (2008). Purification and characterization of glycerolipid acyl-hydrolase from the red alga gracilaria vermiculophylla. Fish. Sci. 74 (3), 670–676. 10.1111/j.1444-2906.2008.01573.x

[B62] Nina S. Ludmila D. Natalia C. Eduard K. Valery S. (2017). Modulation of immunogenicity and conformation of ha1 subunit of influenza a virus h1/n1 hemagglutinin in tubular immunostimulating complexes. Int. J. Mol. Sci. 18 (9), 1895. 10.3390/ijms18091895 PMC5618544 28869526

[B63] Ogunsina M. Pan H. Samadder P. Arthur G. Schweizer F. (2013). Structure activity relationships of N-linked and diglycosylated glucosamine-based anti-tumor glycerolipids. Molecules 18 (12), 15288–15304. 10.3390/molecules181215288 24335578 PMC6270653

[B64] Parveez Ahamed A. A. Rasheed M. U. Peer Muhamed Noorani K. Reehana N. Santhoshkumar S. Mohamed Imran Y. M. (2017). *In vitro* anti-bacterial activity of MGDG-palmitoyl from Oscillatoria acuminata NTAPC05 against extended-spectrum β-lactamase producers. J. Antibiot. 70 (6), 754–762. 10.1038/ja.2017.40 28377637

[B65] Perez Gutierrez R. M. Lule P. R. (2005). Cytotoxic constituents from *Daphnia pulex* . Pharm. Biol. 43 (4), 313–316. 10.1080/13880200590951702 28925836

[B66] Pinto C. Ibáñez M. R. Loyola G. León L. Salvatore Y. González C. (2021). Characterization of an agarophyton chilense oleoresin containing PPARγ natural ligands with insulin-sensitizing effects in a C57Bl/6J mouse Model of diet-induced obesity and anti-oxidant activity in *Caenorhabditis elegans* . Nutrients 13 (6), 1828. 10.3390/nu13061828 34071972 PMC8227508

[B67] Plouguerné E. da Gama B. A. P. Pereira R. C. Barreto-Bergter E. (2014). Glycolipids from seaweeds and their potential biotechnological applications. Front. Cell. Infect. Microbiol. 4, 174. 10.3389/fcimb.2014.00174 25566511 PMC4269193

[B68] Plouguerné E. de Souza L. M. Sassaki G. L. Cavalcanti J. F. Villela Romanos M. T. da Gama B. A. (2013). Antiviral sulfoquinovosyldiacylglycerols (SQDGs) from the Brazilian Brown seaweed sargassum vulgare. Mar. Drugs 11 (11), 4628–4640. 10.3390/md11114628 24284427 PMC3853750

[B69] Plouguerné E. De Souza L. M. Sassaki G. L. Hellio C. Trepos R. Da Gama B. A. (2020). Acidification in the U.S. Southeast: Causes, potential consequences and the role of the southeast ocean and coastal acidification network. Front. Mar. Sci. 7, 1–548. 10.3389/fmars.2020.00548 32802822 PMC7424514

[B70] Prabhakar L. Dicky John D. G. Singh S. R. Murali A. (2022). Computational analysis of marine algal compounds for obesity management against pancreatic lipase. J. Biomol. Struct. Dyn. 1, 1–10. 10.1080/07391102.2022.2074139 35575483

[B71] Qiu S. Khan S. I. Wang M. Zhao J. Ren S. Khan I. A. (2020). Chemometrics-assisted identification of anti-inflammatory compounds from the green alga *Klebsormidium flaccidum* var. Molecules 25 (5), 1048. 10.3390/molecules25051048 PMC7179104 32110943

[B72] Ramm W. Schatton W. Wagner-Döbler I. Wray V. Nimtz M. Tokuda H. (2004). Diglucosyl-glycerolipids from the marine sponge-associated Bacillus pumilus strain AAS3: Their production, enzymatic modification and properties. Appl. Microbiol. Biotechnol. 64 (4), 497–504. 10.1007/s00253-003-1471-8 14593508

[B73] Rath P. D. Chen D. Y. Gu J. Lee V. Al Ani N. A. Shirazy K. (2019). Anti-tumor necrosis factor biosimilars and intended copies in rheumatology: Perspective from the Asia Pacific region. Int. J. Rheum. Dis. 22 (1), 9–24. 10.1111/1756-185X.13371 30338644

[B74] Saadaoui I. Rasheed R. Abdulrahman N. Bounnit T. Cherif M. Jabri H. A. (2020). Algae-derived bioactive compounds with anti-lung cancer potential. Mar. Drugs 18 (4), E197. 10.3390/md18040197 32276401 PMC7230368

[B75] Schmid-Siegert E. Stepushenko O. Glauser G. Farmer E. E. (2016). Membranes as structural anti-oxidants: Recycling of malondialdehyde to its source in oxidation-sensitive chloroplast fatty acids. J. Biol. Chem. 291 (25), 13005–13013. 10.1074/jbc.M116.729921 27143359 PMC4933218

[B76] Shirahashi H. Murakami N. Watanabe M. Nagatsu A. Sakakibara J. Tokuda H. (1993). Isolation and identification of anti-tumor-promoting principles from the fresh-water cyanobacterium Phormidium tenue. Chem. Pharm. Bull. 41 (9), 1664–1666. 10.1248/cpb.41.1664 8221980

[B77] Sun Z. Thilakavathy K. Kumar S. S. He G. Liu S. V. (2020). Potential factors influencing repeated SARS outbreaks in China. Int. J. Environ. Res. Public Health 17 (5), 1633. 10.3390/ijerph17051633 PMC7084229 32138266

[B78] Takahashi Y. Itoh K. Ishii M. Suzuki M. Itabashi Y. (2002). Induction of larval settlement and metamorphosis of the sea urchin strongylocentrotus intermedius by glycoglycerolipids from the green alga ulvella lens. Mar. Biol. 140 (4), 763–771. 10.1007/s00227-001-0749-6

[B79] Terasaki M. Itabashi Y. (2003). Glycerolipid acyl hydrolase activity in the Brown alga Cladosiphon okamuranus TOKIDA. Biosci. Biotechnol. Biochem. 67 (9), 1986–1989. 10.1271/bbb.67.1986 14519986

[B80] Tokuda H. Nishino H. Shirahashi H. Murakami N. Nagatsu A. Sakakibara J. (1996). Inhibition of 12-O-tetradecanoylphorbol-13-acetate promoted mouse skin papilloma by digalactosyl diacylglycerols from the fresh water cyanobacterium Phormidium tenue. Cancer Lett. 104 (1), 91–95. 10.1016/0304-3835(96)04237-1 8640752

[B81] Ulivi V. Lenti M. Gentili C. Marcolongo G. Cancedda R. Descalzi Cancedda F. (2011). Anti-inflammatory activity of monogalactosyldiacylglycerol in human articular cartilage *in vitro*: Activation of an anti-inflammatory cyclooxygenase-2 (cox-2) pathway. Arthritis Res. Ther. 13 (3), R92. 10.1186/ar3367 21682897 PMC3218907

[B82] Wang F. Ding D. Li J. He L. Xu X. Zhao Y. (2020). Characterisation of genes involved in galactolipids and sulfolipids metabolism in maize and arabidopsis and their differential responses to phosphate deficiency. Funct. Plant Biol. 47, 279–292. 10.1071/FP19082 32130107

[B83] Yan H. Xue Z. Xie J. Dong Y. Ma Z. Sun X. (2019). Toxicity of carbon nanotubes as anti-tumor drug carriers. Int. J. Nanomedicine 14, 10179–10194. 10.2147/IJN.S220087 32021160 PMC6946632

[B84] Zhang H. Jiang F. Zhang J. Wang W. Li L. Yan J. (2022). Modulatory effects of polysaccharides from plants, marine algae and edible mushrooms on gut microbiota and related health benefits: A review. Int. J. Biol. Macromol. 204, 169–192. 10.1016/j.ijbiomac.2022.01.166 35122806

[B85] Zhang J. Li C. Yu G. Guan H. (2014). Total synthesis and structure-activity relationship of glycoglycerolipids from marine organisms. Mar. Drugs 12 (6), 3634–3659. 10.3390/md12063634 24945415 PMC4071594

[B86] Zhang W. Huang Q. Xiao W. Zhao Y. Pi J. Xu H. (2020). Advances in anti-tumor treatments targeting the CD47/SIRPα Axis. Front. Immunol. 11, 18. 10.3389/fimmu.2020.00018 32082311 PMC7003246

[B87] Zhang X. Wang N. Wang Z. Liu Q. (2020). The discovery of segmented flaviviruses: Implications for viral emergence. Curr. Opin. Virol. 40, 11–18. 10.1016/j.coviro.2020.02.001 32217446

